# Dental Instruction

**Published:** 1888-09

**Authors:** 


DENTAL INSTRUCTION.—We are authoritatively informed that the faculty of the Dental Department of the Southern Medical College are making preparations for a large class in dentistry. The college opens on the second day of October next. The students of this institution will be well and thoroughly instructed in every department. The students are permitted the full benefits of the Lectures and Demonstrations in Anatomy and Physiology as provided for the medical class, and for a matriculation fee of $5.00 secures the privilege also of attending the lectures in the other departments of medicine. Persons desiring particular information in regard to this excellent school should

Address, DR. L. D. CARPENTER, *Dean*,

47½ Whitehall st., Atlanta, Ga.

